# Examination of mechanisms (E-MECHANIC) of exercise-induced weight compensation: study protocol for a randomized controlled trial

**DOI:** 10.1186/1745-6215-15-212

**Published:** 2014-06-07

**Authors:** Candice A Myers, William D Johnson, Conrad P Earnest, Jennifer C Rood, Catrine Tudor-Locke, Neil M Johannsen, Shannon Cocreham, Melissa Harris, Timothy S Church, Corby K Martin

**Affiliations:** 1 Pennington Biomedical Research Center, 6400 Perkins Road, Baton Rouge, LA 70808, USA; 2 School of Kinesiology, College of Human Sciences & Education, Louisiana State University, 112 Long Fieldhouse, Baton Rouge, LA 70803, USA

**Keywords:** Compensation, Energy expenditure, Energy intake, Exercise training, Randomized controlled trial, Weight loss

## Abstract

**Background:**

Weight loss induced only by exercise is frequently less than expected, possibly because of compensatory changes in energy intake and/or energy expenditure. The purpose of the Examination of Mechanisms (E-MECHANIC) of Exercise-Induced Weight Compensation trial is to examine whether increased energy intake and/or reduced spontaneous activity or energy expenditure (outside of structured exercise) account for the less than expected, exercise-associated weight loss.

**Methods/Design:**

E-MECHANIC is a three-arm, 6-month randomized (1:1:1) controlled trial. The two intervention arms are exercise doses that reflect current recommendations for (1) general health (8 kcal/kg body weight per week (8 KKW), about 900 kcal/wk) and (2) weight loss (20 KKW, about 2,250 kcal/wk). The third arm, a nonexercise control group, will receive health information only. The sample will include a combined total of 198sedentary, overweight or obese (body mass index: ≥25 kg/m^2^ to ≤45 kg/m^2^) men and women ages 18 to 65 years. The exercise dose will be supervised and tightly controlled in an exercise training laboratory. The primary outcome variables are energy intake, which will be measured using doubly labeled water (adjusted for change in energy stores) and laboratory-based food intake tests, and the discrepancy between expected weight loss and observed weight loss. Secondary outcomes include changes in resting metabolic rate (adjusted for change in body mass), activity levels (excluding structured exercise) and body composition. In an effort to guide the development of future interventions, the participants will be behaviorally phenotyped and defined as those who do compensate (that is, fail to lose the amount of weight expected) or do not compensate (that is, lose the amount of weight expected or more).

**Discussion:**

In this study, we will attempt to identify underlying mechanisms to explain why exercise elicits less weight loss than expected. This information will guide the development of interventions to increase exercise-induced weight loss and maximize weight loss retention and related health benefits.

**Trial registration:**

ClinicalTrials.gov ID: NCT01264406 (registration date: 20 December 2010).

## Background

The prevalence of obesity is increasing worldwide. It has become an epidemic and is associated with many chronic diseases [1,2]. Although exercise is commonly cited as an important strategy for weight reduction, researchers in numerous well-controlled and statistically well-powered studies have demonstrated that exercise training without a dietary intervention results in far less weight loss than expected, given the expended calories. This is most evident when the exercise dose is substantial (≥60 min/day) [3]. In studies of regimens lasting 26 weeks or more (n = 12), researchers determined that there was no dose–response relationship between prescribed energy expenditure and weight loss and that actual weight loss was only 30% of what was expected [3].

The potential explanations for the failure of exercise training to produce the expected weight loss in long-term studies include poor exercise regimen adherence, reduced spontaneous activity, reduced resting metabolic rate and/or increase in energy (that is, food) intake. The results of well-controlled studies do not suggest that poor adherence is the source of the discrepancy between actual and expected weight loss. For example, Donnelly *et al*. examined the role of supervised exercise in weight loss over the course of 16 months in college-aged men and women [4]. The required exercise energy expenditure was 3,300 kcal/wk for men and 2,200 kcal/wk for women. Only the men lost weight (mean = −5.2 kg) compared to their control group peers, but the mean 5.2-kg weight loss was only 19% of what was expected (64 weeks × 3,300 kcal/wk)/7,700 kcal/kg = 27.4 kg), given the dose of exercise. Exercising women maintained their weight, despite expending 2,200 kcal/wk during exercise, and the nonexercising female controls gained weight. On the basis of these results, the researchers suggested that increased energy intake, rather than decreases in resting metabolic rate or spontaneous physical activity, might have contributed to the less-than-expected weight loss [4-6].

Researchers who have examined engaging in exercise for weight loss have found equivocal results showing less-than-expected changes in body weight, particularly when associated with high doses of exercise, with relatively higher interindividual and between-sexes variability [7]. The mechanisms responsible for the failure of exercise to produce substantial weight loss in most, but not all, individuals need to be elucidated. A better understanding of the mechanisms responsible for this compensation is critical to developing strategies to optimize the health benefits associated with exercise [8]. Blundell *et al*. noted that a large amount of variability in the degree to which people increase energy intake in response to exercise. Hence, people can be categorized as “compensators” and “noncompensators,” in this regard; yet, little is known about the characteristics of these groups or the mechanisms responsible for compensation [9]. In accord with this, King *et al*. emphasized the need to examine weight compensation in response to exercise and develop individualized interventions in order to prevent less-than-expected weight loss [10]. These researchers indicated that a fundamental criticism of engaging in exercise for weight loss is indeed the issue of compensation. The energy deficit caused by exercise is counteracted by compensatory behaviors, and thus weight control is hindered. These authors outlined the components of energy intake and expenditure that might volitionally or nonvolitionally change in response to exercise, including metabolic and behavioral factors. Furthermore, the authors reported that important limitations of the existing research include the short duration of exercise interventions, measurement of energy intake for short periods of time and difficulty in obtaining accurate measurements of energy intake and expenditure.

The US Department of Health and Human Services *Physical Activity Guidelines Advisory Committee Report*[11], in conjunction with information provided by most other government-based health institutions and professional health associations, include a recommendation of at least 150 minutes of moderate-intensity aerobic physical activity per week to improve overall health. In a report published by the Institute of Medicine, the US National Research Council concluded that an exercise regimen of 60 minutes of moderate-intensity physical activity per day, or a total of 300 minutes per week, is necessary to prevent unhealthy weight gain [12]. The 2005 US Department of Agriculture (USDA) dietary guidelines also suggest that a regimen of 60 minutes or more of daily activity is required to prevent weight gain [13]. Although no consensus exists regarding the amount of physical activity required to lose weight, it is generally recommended that overweight and obese individuals increase their daily physical activity to 200 to 300 min/wk (45 to 60 min/day) or ≥2,000 kcal/wk. Given the paucity of literature supporting these recommendations, further empirical evidence is needed to understand potential compensatory mechanisms that may impede healthy weight loss and weight loss retention.

An important limitation of the extant literature is the overall short duration of the exercise interventions and the general lack of accuracy of measurements of energy balance (that is, energy intake and energy expenditure) [10]. Further, US national guidelines and recommendations cite specific physical activity goals for health improvement, but this advice is not informed by rigorous research results and does not acknowledge the potential for counterproductive compensatory mechanisms. The Examination of Mechanisms (E-MECHANIC) of Exercise-Induced Weight Compensation trial will directly address these issues. The aim of the E-MECHANIC trial is to test two doses of exercise and their effect on change in all aspects of energy balance, including energy intake, metabolism, physical activity and body composition, using the most precise assessment methods available. The E-MECHANIC investigators hope to effectively determine the source, or sources, of alterations in energy balance that reduce the magnitude of weight loss during an exercise program. In addition, the study researchers intend to determine the behavioral phenotypes associated with participants who do (noncompensators) and those who do not (compensators) lose the expected amount of weight in response to an increase in energy expenditure through exercise.

## Methods/Design

### Institutional Review Board approval and trial registration

The study protocol was approved by the Institutional Review Board of the Pennington Biomedical Research Center (Baton Rouge, LA, USA; protocol 10008). The ClinicalTrials.gov identifier is NCT01264406.

### Study design and objectives

The E-MECHANIC study is a three-arm, 6-month randomized controlled trial (RCT). The selected exercise doses reflect current recommendations for (1) general health (8 kcal/kg body weight/wk (8 KKW), or approximately 800 to 1,000 kcal/wk) and (2) weight loss (20 KKW, or approximately 2,000 to 2,500 kcal/wk) [14]. A nonexercise control group will also be included in the study.

The primary objectives of the E-MECHANIC trial are (1) to test whether energy intake increases in response to either or both of two doses of exercise (8 KKW vs. 20 KKW), (2) to test whether the discrepancy between observed and expected weight loss (Wt Loss_diff_) differs between the two exercise groups (8 KKW vs. 20 KKW) and between the control and 20-KKW groups and (3) to determine whether change in energy intake mediates Wt Loss_diff_.

The secondary objectives of the trial are (1) to determine whether change in resting metabolic rate (RMR; adjusted for change in body mass index (BMI)) and activity levels (excluding structured exercise) differ across treatment groups; (2) to determine whether change in body composition differs by exercise dose; and (3) to characterize participants who compensate (that is, fail to lose the amount of weight expected) and compare them with those who do not compensate (that is, lose the amount of weight expected or more).

### Participants

The recruitment goal in the E-MECHANIC trial is a combined total of 198 men and women. The inclusion criteria are being sedentary (that is, not exercising for more than 20 minutes at least 3 days per week based on self-report and 1 week of accelerometer data) and being overweight or obese (BMI ranging from ≥25 kg/m^2^ to ≤45 kg/m^2^). The exclusion criteria are current consumption of more than 14 alcoholic drinks per week, smoking within the past 6 months, pregnancy, having been pregnant within the past 6 months, breastfeeding, history of weight loss surgery (if a participant had a gastric band that was removed, the participant may be eligible at the discretion of the Principal Investigator (PI)), current participation in a weight loss program, medical condition such as diabetes and cardiovascular disease (CVD; potential participants with a history of CVD who are under the care of a physician who is treating the CVD will be considered for enrollment in the study) and inability to complete the study within the designated time frame because of plans to move out of the study area.

### Assessments and schedule of procedures

The E-MECHANIC trial will have three assessment periods during which procedures to collect data for the primary and secondary study outcomes will be carried out. The primary outcomes are (1) energy intake and (2) Wt Loss_diff_. Energy intake will be measured using two methods: doubly labeled water (DLW) and laboratory-based food intake tests. The secondary outcomes are changes in RMR, objectively measured activity levels (excluding structured exercise) and body composition. The primary assessments will occur at baseline and at week 24, with identical procedures followed at both assessment periods, and a truncated assessment period at week 4 to allow for identification of potential short-term changes in the study outcomes. Table 1 outlines the schedule of the E-MECHANIC study procedures.

**Table 1:** **Schedule of study procedures**^**a**^

**Evaluations and procedures**		**Baseline**	**Week 4**	**Week 24**
	**Screening**	**(day −14 to day 0)**	**(day 28 to day 35)**	**(day 160 to day 174)**
Informed consent and HIPAA authorization	OR, SV1			
Randomization		Day 0		
Anthropometrics and body composition				
Height	RI1, SV1			
Waist and hip circumference	SV1		Day 28	Days 160, 174
Weight	RI1, SV1	Days −14, −7, 0	Days 28, 35	Days 160, 167, 174
Blood pressure and heart rate	RI1, SV1		Day 28	Days 160, 174
ECG	SV1			Day 160
DXA		Day −14, 0		Days 160,174
Energy metabolism				
TEE based on DLW measurement		Day −14		Day 160
Daily weight		Day −14 to day 0		Day 160 to day 174
Urine collection		Days −14, −7, 0		Days 160, 167, 174
RMR		Day −14		Day 160
Physical activity				
Accelerometry	RI2	Day −14	Day 28	Day 160
VO_2_max	SV2			Day 163
Psychological questionnaires^b^		Day −14	Day 28	Day 160
Clinical chemistry panel (CBC, chem 15)	SV1			Day 160
Food intake tests with VAS		Day −4	Day 28	Day 170
Blood collection for satiety hormone archive^c^		Day −4		Day 170

^a^CBC, Complete blood count; chem 15, Panel of laboratory tests measuring 15 blood chemistry components; DLW, Doubly labeled water; DXA, Dual-energy X-ray absorptiometry; ECG, Electrocardiography; HIPAA, Health Insurance Portability and Accountability Act; OR, Orientation; RI, Run-in; RMR, Resting metabolic rate; SV, Screening visit; TEE, Total energy expenditure; VAS, Visual Analogue Scale; VO_2_max, Maximal oxygen uptake. ^b^The Psychological questionnaires to be used are the Activity Temperament Questionnaire, Body Morph Assessment, Compensatory Health Beliefs Scale, Eating Inventory, Food Craving Inventory, Food Preference Questionnaire, Medical Outcomes Study 36-item Short Form, Multifactorial Assessment of Eating Disorder Symptoms, Pittsburgh Sleep Quality Index, Retrospective Visual Analogue Scale, Yale Food Addiction Scale. ^c^Before and after food intake tests.

The trial will include a multistage screening process prior to participant enrollment consisting of a 3- to 4-week period that includes 1 week of run-in. Following an initial web and/or telephone screen to determine eligibility and provide the potential participants with a brief description of the study, the potential participants will be scheduled for an orientation session where detailed information about the study will be provided. The detailed information will include the number and type of assessments, the length and nature of the exercise training and the time commitment required to complete the study. Next, potential participants will undergo three run-in visits meant to reduce attrition by allowing them to determine if they will be able to schedule the study into their weekly routine. All three run-in sessions must be completed within the 1-week period to satisfy the eligibility criteria. During screening, informed consent will be obtained from each eligible participant by study staff. Staff members will also verify that eligible participants are not currently meeting public health physical activity guidelines (that is, sedentary: <8,000 steps/day over the course of 1 week) [15] by having them wear SenseWear Armband (BodyMedia, Pittsburgh, PA, USA) accelerometers.

After being screened into the trial, participants will complete the comprehensive baseline assessment and will be randomly assigned to one of the exercise arms (8 KKW or 20 KKW) or to the control group (nonexercise arm). Participants will be enrolled at a 2:1 female/male ratio and randomized separately to one of the three intervention groups at a 1:1:1 ratio to obtain an equal number of women and men in each treatment group. Participants who drop out (that is, fail to return for the 6-month examination) will not be replaced. Participants in the 8-KKW and 20-KKW intervention groups will continue to attend exercise sessions during week 4 and week 24 assessment periods. Participants who have not met the intervention energy expenditure goals by week 24 will be allowed to exercise up to 3 weeks after the week 24 assessment period.

### Interventions

The intervention will commence within 48 hours after a participant’s randomization and span 24 weeks.

#### Control group

Participants assigned to the control group will receive multimedia health information twice weekly by text messaging or e-mail throughout the study period. The health information covers a variety of topics, including stress management and the benefits of eating fruits and vegetables [16-19]. Monthly seminars will be available for control group participants and will cover topics related to a healthy lifestyle. In addition, they will be sent a quarterly newsletter that features information on healthy living. During the 6 months of study participation, control group participants will be instructed to maintain their baseline level of physical activity. After the 6-month study is complete, control group participants will have the option of exercising in the fitness center at Pennington Biomedical Research Center for eight sessions within 1 month after their last assessment visit (that is, day 174). The fitness center staff will be available to provide instruction and information on an appropriate exercise prescription. In addition, control group participants will be given an opportunity to meet with a dietitian following the intervention.

#### Exercise protocols for the 8-KKW and 20-KKW groups

In the 8-KKW and 20-KKW groups, the exercise intensity for each participant will be set at a target heart rate associated with 65% to 85% of peak oxygen uptake (VO_2peak_). All exercise training will occur on a treadmill. The exercise prescription will initially be based on peak oxygen uptake determined by giving participants a graded treadmill exercise test. In brief, the treadmill speed will start at 2.4 mph on a level grade for 2 minutes, and then increases in speed and/or grade will be applied to produce about a 1 metabolic equivalent of task (MET) increase in workload every 2 minutes until volitional fatigue is reached. During exercise training sessions, the speed and grade of the treadmill will be altered to keep the participants within their target heart rate range (65% to 85% of VO_2peak_), and the participants will be allowed to select their exercise intensity within that range. On the basis of speed, grade, participant’s weight and standard American College of Sports Medicine (ACSM) equations, energy expenditure will be calculated in real time and the length of each exercise session will be adjusted to meet the daily caloric goal [12,20]. The caloric goal of each session will be calculated by dividing the prescribed exercise dose (8 KKW or 20 KKW) by the participant-selected exercise frequency (that is, three, four or five times per week).

In order to precisely match the caloric exercise goals of the study, we will measure actual energy expenditure (AEE) while exercising and appropriately adjust exercise time. Within the first week of the exercise intervention and once every 2 to 4 weeks thereafter for the remainder of the intervention, trained personnel will measure caloric expenditure rate using a metabolic cart while the participant is walking on a treadmill at a predetermined speed and grade. The metabolic cart data will be used to (1) verify the accuracy of the ACSM equations on an individualized basis, (2) adjust the participant’s daily exercise time to account for changes in metabolic or biomechanical efficiency that may occur due to the exercise training and (3) ensure that calories carried over from previous weeks remain equivalent throughout the study. By correcting the daily exercise time, we can precisely increase or decrease the amount of calories expended per session to match the prescribed weekly exercise AEE.

During exercise training sessions, exercise intensity will be monitored by using a heart rate signal from a Polar transmitter placed around the participant’s chest (Polar Electro, Lake Success, NY, USA) and recorded every 5 minutes along with the participant’s subjective rating of perceived exertion using the Borg Rating of Perceived Exertion scale. Participants will be allowed to read, listen to music, watch television or socialize with study staff while they exercise. The training program consists of a 3-minute warm-up at a standard intensity level, and then the prescribed training intensity will be initiated. We expect that most participants randomized to the 8-KKW group will be capable of exercising at their required dose during the first week of exercise. Participants randomized to the 20-KKW group may not be able to complete their assigned dose of exercise immediately upon entry into the study. In the 20-KKW group, the exercise dose will progress from 8 KKW during the first week, to 14 KKW during the second week and ultimately to the full prescription (20 KKW) during the third week. By the end of the third week, participants will be expected to perform 100% of their weekly exercise dose for 3 or 4 days in the 8-KKW group and for 3 to 5 days in the 20-KKW group.

Table 2 shows the estimated number of calories and minutes per week required by participants to expend 20 KKW. The number of calories and minutes required per session for each exercise dose is shown for those assigned the weekly dose in 3, 4 or 5 days. The flexibility of allowing participants to choose the number of days per week that they would like to exercise allows for preferred differences in the length of exercise sessions. We have found that this flexibility helps with participant compliance and overall satisfaction.

**Table 2:** **Estimates of energy expenditure and exercise duration**^**a**^

	**3 sessions/wk**		**4 sessions/wk**		**5 sessions/wk**	
**Exercise regimen**	**TEE**	**Minutes**	**TEE**	**Minutes**	**TEE**	**Minutes**
Treadmill (3.2 mph), 5% grade	667 kcal	92	500 kcal	69	400 kcal	55
Treadmill (4 mph), 5% grade	667 kcal	80	500 kcal	62	400 kcal	50

^a^TEE, Total energy expenditure. Estimates given are the energy expenditure in kilocalories and exercise duration at a heart rate associated with 65% peak oxygen uptake necessary for a 100-kg participant to achieve 20.0 kcal • kg^-1^ • wk^-1^ energy expenditure.

### Primary study outcome measures

#### Energy intake

During the E-MECHANIC trial, we will rely on two complementary, state-of-the-art (“gold standard”) methods to measure energy intake: DLW and laboratory-based food intake tests. Energy intake will be measured twice during 2-week periods at baseline (days −14 to 0) and week 24 (days 160 to 174) with DLW, which is used to obtain an accurate measure of daily total energy expenditure (TEE). During a period of energy balance, TEE is equal to energy intake [21,22]. When energy balance is not present during the DLW assessment period, adjustments will be made for the deviation in energy balance as determined by change in body mass (energy stores) [23,24]. Body weight will be assessed by measuring metabolic weight under fasting conditions in the clinical assessment laboratory at three time points (days −14, −7 and 0 for baseline; days 28 and 35 for week 4; and days 160, 167 and 174 for week 24). Participants will also record their daily body weight at home during the DLW period with equipment provided by study staff. Clinic weights and daily home-based weights will be utilized to adjust TEE for deviation in energy balance. A strength of the DLW method is its accuracy for measuring energy expenditure, and thus energy intake, in free-living conditions [21,23-25]. Measuring food intake with DLW is not associated with significant undereating or weight change [26,27], suggesting that this is the ideal method of testing for compensation or increased energy intake in response to exercise.

Because DLW cannot quantify macro- and micronutrient intake (DLW provides only a measure of energy or kilocalorie intake), we will quantify energy and nutrient intake in controlled conditions using laboratory-based food intake tests. The strengths of this method include reliability (that is, accurate replication) of food intake measurements and the ability to empirically test the effect of interventions on food intake [28]. Food and nutrient intake during the laboratory tests have been shown to be representative of habitual food intake and similar to that measured in cafeteria settings [29,30]. Hence, laboratory-based food intake tests are an ideal method of quantifying energy and nutrient intake, particularly when the study design includes a measure of energy (that is, caloric) intake collected in free-living conditions (that is, DLW).

The food intake tests will occur in a laboratory under controlled conditions at baseline (day −4), week 4 (day 28) and week 24 (day 170). On the morning of food intake testing, participants will be instructed to consume a provided 190-kcal nutrition bar between 7:00 AM and 8:00 AM. Each participant will consume the same breakfast item on all three food intake test days. Participants will enter the laboratory between 11:00 AM and 12:00 PM to complete a test lunch, which will consist of *ad libitum* sandwiches, potato chips, cookies, water and choice of artificially sweetened or sugar-sweetened soda or tea. Participants will then return 5.5 hours after the start of their lunch to complete their dinner meal, which will consist of a buffet meal of 18 high- and low-fat foods as previously described [31]. Week 24 food intake assessments will be scheduled at least 24 hours after the most recent exercise session. Food intake during lunch and dinner will be precisely quantified by weighing food provision and plate waste to the 0.1-gram level. Participants will be blinded to the procedures and the purpose of food testing. Nutrient information will be calculated using the USDA’s Food and Nutrient Database for Dietary Studies (FNDDS) [32]. We will use computer software that is programmed with these standards to calculate and record food intake parameters, including the amount (in grams) and energy content (in kilocalories) of consumed food. In addition, macronutrient intake will be calculated using FNDDS, which will allow us to quantify (1) the grams of protein, carbohydrate and fat (saturated and unsaturated) eaten; (2) the kilocalories of energy obtained from these macronutrients; and 3) the percentage of kilocalories obtained from protein, carbohydrate and fat (saturated and unsaturated). The computer program also provides a complete breakdown of micronutrients, vitamins and minerals (for example, intake of fiber, sodium, vitamin A, calcium and iron).

#### Difference between expected weight loss and observed weight loss

Observed weight loss will be calculated based on the difference in body weight from baseline to follow-up. Expected weight loss will be calculated using two methods. The first method assumes that 1 kg of weight represents 7,700 kcal of energy. Calories expended during supervised exercise is divided by 7,700 kcal to derive expected weight loss (exercise energy expenditure divided by 7,700 kcal/kg) [33]. The second method is based upon more recent research which demonstrates that a 7,700-kcal deficit does not always produce a 1 kg of weight loss, because this formula overestimates weight loss [34-36]. The researchers who produced this body of work developed a more accurate model that accounts for the dynamics of weight change during exercise. This dynamic formula will be used to calculate expected weight loss, given the two different doses of exercise, and the accompanying observed weight loss. Weight loss difference will then calculated as observed weight loss minus expected weight loss [33].

### Secondary study outcome measures

#### Resting metabolic rate

Indirect calorimetry will be used to measure RMR on day −14 and day 160. Energy expenditure will be adjusted for change in BMI, as outlined by Martin *et al*. [37,38]. Each participant’s resting metabolism will be measured over a 30-minute period after a 12-hour overnight fast using a MAX-II metabolic cart system (AEI Technologies, Pittsburgh, PA, USA). After the participant has quietly rested for 30 minutes, a transparent plastic hood connected to the cart will be placed over the participant’s head. For the duration of the test, the participant will be asked to remain motionless and awake. Prior to each measurement, the pneumotach flowmeter will be calibrated using a 3-L calibration syringe (Hans Rudolph Inc, Shawnee, KS, USA), and gas analyzers will be calibrated using standardized gas mixtures. The inspired gas samples will be diluted by flow adjustment to maintain a carbon dioxide concentration between 0.7% and 0.9%. Oxygen uptake and carbon dioxide production will be calculated using M-II software. The average of the last 20 minutes of the measurement will be used to calculate RMR using Weir equations [39].

#### Activity level

SenseWear armbands will be used to measure physical activity and sedentary behavior at multiple time points throughout the study, including screening, the 2-week DLW period during baseline, week 4 and week 24. The armband is a small device that fits on the upper arm and has five types of sensors that continuously record data: a two-axis accelerometer, two galvanic skin response sensors, a heat flux sensor, a skin temperature sensor and a near-body temperature sensor. Body weight, height, handedness, age, sex and smoking status will be used in the calculation of energy expenditure, which will be carried out using proprietary software. The armband also records the length of time that it was worn on the body, which will allow us to detect noncompliance with data collection procedures. Activity data will be summated per day and displayed in 1-minute epochs, allowing data from days with poor compliance to be imputed or eliminated from the statistical analyses. Participants will be instructed to wear the armband 24 hours/day and remove it while showering, bathing or swimming. The participants randomized to one of the two exercise groups will be instructed to wear the armband during the exercise sessions. If the participant is <95% compliant with wearing the armband during screening or week 4 of testing, they will be asked to wear the armband for an additional 7 days. During the midpoint of baseline (day −7) and week 24 of testing (day 167), the participant’s armband will be checked for compliance. If found to be noncompliant, the participant will be counseled by study staff about the importance of wearing the armband as directed. Participants can be excluded prior to randomization for armband noncompliance.

In addition to quantifying duration on the body, the SenseWear armband records key aspects of sedentary and active behaviors, including components of posture allocation, such as (1) number of minutes lying down per day, (2) number of minutes of sleep per day, (3) number of steps taken per day and (4) minutes per day spent in activities of different intensities (sedentary, moderate, vigorous and very vigorous). The armband quantifies activity intensity on the basis of MET cut points; for example, ≤3.0 METs is classified as sedentary behavior. The armband also quantifies (1) TEE, (2) RMR, (3) activity energy expenditure (AEE) (4) mean MET cut point and (5) minutes per day spent in physical activity. Importantly, the armband quantifies aspects of active and sedentary behaviors not captured by other methods, namely, DLW and RMR. The armbands will be used to quantify important changes in active and sedentary behaviors in response to exercise (between the 8- and 20-KKW groups and between the control and 20-KKW groups). These armband data will also be used to determine if there have been different changes in active and sedentary behaviors in response to exercise between compensators and noncompensators.

Extensive validity data on the SenseWear armband have been published. These data show that the armband could be used to estimate TEE more accurately than DLW over the course of 10 days in free-living conditions [40] and also estimated RMR more accurately than indirect calorimetry [41]. The armband provides a good measure of the amount of time spent in activity [42], and, of five accelerometers tested, it was the most accurate tool for estimating TEE across different intensities of activity [43]. Other researchers found that the armband provides a valid and reliable measurement of energy expenditure during resting conditions and during different intensities of activity, though variability in measuring energy expenditure increased during activity [44,45].

#### Body composition

Measures of body composition, including fat and lean mass, will be assessed by dual-energy X-ray absorptiometry (DXA) (Lunar iDXA with Encore software version 13.60; GE Healthcare, Madison, WI, USA) at baseline (day −14 and day 0) and week 24 (day 160 and day 174). Lean mass measurements taken with DXA are used to adjust RMR for alterations in body composition [37]. Measurement data from DXA recordings will also be used to explore whether fat or fat-free mass changes differ between treatment groups and between compensators and noncompensators. The use of DXA involves minimal X-ray exposure—about the same as 12 hours of background radiation from the sun.

### Compensators and noncompensators

A secondary objective of the E-MECHANIC trial is to phenotype the behavior of participants in order to characterize compensators and noncompensators. This has been identified previously as an area in need of further study [9]. We carefully selected a battery of self-report instruments to comprehensively measure behavioral constructs associated with energy intake, body image, temperament and health-related quality of life. These questionnaires will be administered at baseline (day −14), week 4 (day 28), and week 24 (day 160). The measurement instruments described in the next two subsections will be used. All of them have been found to be reliable and valid.

#### Measurement of constructs associated with eating attitudes and behaviors

Visual Analogue Scales (VAS) are administered to measure subjective ratings of appetite [46]. Specifically, two forms of VAS data will be collected. First, a VAS will be completed by participants immediately before and after the food intake test meals. Second, retrospective VAS will be used to measure average ratings of satiety that participants experienced during the previous week. This method of collecting VAS data has been found to be consistent with daily assessments of satiety [47]. Supporting evidence for the reliability and validity of VAS data for measuring subjective states related to energy intake has been published [46].

The following instruments will be used to asses constructs associated with eating attitudes and behaviors: (1) the Multifactorial Assessment of Eating Disorders Symptoms (MAEDS), (2) the Eating Inventory (EI), (3) the Food Preference Questionnaire (FPQ), (4) the Food Craving Inventory (FCI) and (5) the Yale Food Addiction Scale (YFAS). The MAEDS is a 56-item measure of eating-disordered behavior that assesses depression, binge eating, purgative behavior, fear of fatness, restrictive eating and avoidance of forbidden foods [48,49]. The EI is designed to measure different dimensions of eating behavior, including cognitive restraint, disinhibition and hunger [48,49]. The FPQ is a self-report measure of food preference, including preference for sugars, complex carbohydrates, protein and fat [50]. The FCI is a measure of specific food craving and assesses the frequency with which an individual experiences craving for a particular food [51]. The YFAS is used to identify individuals who show tendencies toward addictive behaviors for certain types of food, such high-fat and high-sugar foods [52].

#### Measurement of constructs associated with physical activity and overall health

To measure constructs related to the propensity for physical activity and attitudes about general health, a number of valid questionnaires will be administered to participants. First, the tendency to participate in movement throughout the day will be assessed using the Activity Temperament Questionnaire [53,54]. Second, the Medical Outcomes Study 36-item Short Form will be used to measure mental and physical aspects of quality of life [55]. Third, sleep quality and disturbances over a 1-month interval will be assessed with the Pittsburgh Sleep Quality Index. Fourth, the Compensatory Health Beliefs Scale will be used to measure compensatory health-related beliefs, such as justifying eating an excessively large meal because one plans to exercise later in the day [56,57]. Fifth, body image and body dissatisfaction will be measured using the Body Morph Assessment (BMA) computer program [58]. BMA is a system that presents body silhouettes to participants. The body silhouettes range from very thin to very obese, and individuals being assessed manually increase or decrease the size of the silhouette by using a pointer on the computer screen. In this way, they indicate their current body size (CBS), ideal body size (IBS) and realistic body size (RBS). Two discrepancy scores (CBS − IBS and CBS − RBS) are derived as the differences between CBS and IBS and IBS.

### Statistical power and sample size

The power analyses will focus on the primary outcomes, that is, energy intake (measured with DLW and laboratory-based food intake tests) and Wt Loss_diff_. In our power analyses, we will make the following assumptions: (1) power ≥0.90 and above is ideal; (2) the significance level under the null hypothesis will be set at α = 0.05; (3) the power analyses will be based on the sample size expected at the end of the study (that is, after 10% attrition); and (4) null hypotheses will be tested against two-directional (that is, two-tailed) alternative hypotheses. We will rely on conservative estimates of effect size and liberal estimates of variance, as well as the assumption that 1 kg of weight represents 7,700 kcal energy, in our power analyses [33]. We expect no less than 60 participants in each of the three groups to finish the study (8 KKW, 20 KKW and control). The estimated effect sizes are shown in Table 3. They indicate that statistical power is identical for the comparisons between the 8-KKW and 20-KKW groups and between the control and 20-KKW groups.

**Table 3:** **Effect sizes and power calculations for the primary outcome measures**^**a**^

						**Statistical power**	
**Endpoints and comparisons**	**Sample 1**	**Sample 2**	**Differences between groups**	**SD**	**Effect size**	**One-tailed test**	**Two-tailed test**
Energy intake (kcal), DLW							
8 KKW vs. 20 KKW	60	60	200	316	0.63	0.96	0.93
Energy intake (kcal), laboratory							
8 KKW vs. 20 KKW, control vs. 20 KKW	60	60	200	363	0.61	0.90	0.95
Wt Loss_diff_							
8 KKW vs. 20 KKW, control vs. 20 KKW	60	60	2.0	3.3	0.61	0.95	0.90

^a^KKW, Kilocalories per kilogram of body weight per week; Wt Loss_diff_, Discrepancy between expected weight loss and observed weight loss.

#### Energy intake

The power analyses for the two methods by which energy intake will be measured rely on the effect sizes given in Table 3. The power analyses for energy intake, measured on the basis of DLW, indicate that the study will have a power of 0.93 for us to detect a 200-kcal difference in changes in energy intake between the 8-KKW and 20-KKW groups and between the control and 20-KKW groups. The study will also have 0.95 power for us to detect a 200-kcal difference in changes in energy intake measured with food intake tests in the laboratory between the 8-KKW and 20-KKW groups and between the control and 20-KKW groups.

#### Discrepancy between expected weight loss and observed weight loss

The effect sizes for the calculations and the power analyses are summarized in Table 3. They indicate that the study will have a power of 0.90 for the Wt Loss_diff_ comparisons.

### Data analysis

Analytic data will be archived in an electronic file. Prior to locking the file against further change, we will inspect all data for completeness, proper range and internal consistency. This data-cleaning process will include mock analyses by an analyst blinded to intervention group assignment. Any changes made will be documented in permanent records. Statistical analyses will be performed only after careful consideration is given to the assumptions underlying the statistical methods employed, using model diagnostics such as quantile plots of studentized residuals, component-plus-residual plots and examination of leverage points and outliers.

First, baseline participant characteristics (for example, age, sex, race, weight, BMI, waist circumference) will be summarized for each intervention group as counts and percentages for categorical variables and as means and 95% confidence intervals for continuous variables. The statistical significance of differential longitudinal changes in response to interventions will be assessed by employing repeated-measures mixed-effects models with maximum likelihood estimation and Kenward-Roger adjustment to the degrees of freedom of the relevant test statistic. The three interventions will be compared across assessment times (for example, baseline, week 4, week 24). Intervention, race, sex and assessment times will be taken as fixed effects. Participants within groups will be considered as having random effects. Covariates such as age and baseline assessments will be included in preliminary models and retained in the final analytic models if warranted. The model covariance structure (for example, unstructured, compound symmetric, autoregressive) across time will be investigated to enhance the efficiency of the statistical tests. The results for each outcome variable will be summarized as least-squares means and 95% confidence intervals for each intervention group across the assessment times. Model residuals will be tested to see if distributions are approximately Gaussian and data transformations (for example, logarithmic) will be performed if needed. Group differences in baseline characteristics will be tested, and corrective steps, such as poststratification and addition of model covariates, will be taken if necessary. Linear contrasts on least-squares means will be used to test pairwise differential mean changes in each outcome measure from baseline to subsequent assessment times between the three intervention groups. Null hypotheses will be tested against two-directional alternatives at the nominal 0.05 significance level using Tukey-Kramer–adjusted *P*-values when appropriate. All statistical analyses will be performed using SAS version 9.3 statistical software (SAS Institute, Cary, NC, USA).

Second, we will characterize compensators vs. noncompensators, where *compensators* will be defined as those who fail to lose the expected amount of weight during the exercise trial and *noncompensators* will be defined as those who meet or exceed the expected amount of weight loss during the trial. Differences between the groups on the following variables will be tested: (1) baseline values derived from self-report instruments, (2) baseline energy intake measures (for example, total kilocalories, macronutrient intake and fat and sugar intake) and (3) baseline activity levels (quantified by SenseWear armband readings). In addition, differences between compensators and noncompensators will be evaluated for sex and race disparities, and compensators and noncompensators will be compared with respect to changes in outcomes from baseline to week 24.

As an initial step in a mediator analysis, we will test whether changes in energy intake mediate or partially mediate the relationship between group assignment (exercise dose) and Wt Loss_diff_. We will follow the methods of Baron and Kenny [59] and determine whether Wt Loss_diff_ is predicted by group, with grouping variables coded as 8 KKW vs. 20 KKW and control vs. 20 KKW. Subsequently, we will test whether change in energy intake predicts Wt Loss_diff_. Assuming that the results are significant, we will then determine whether group significantly predicts Wt Loss_diff_ independently of energy intake. If group does not independently predict Wt Loss_diff_, then the relationship between group and Wt Loss_diff_ may be attributable to change in energy intake. That is, we will then conclude that change in energy intake mediated the relationship between group and Wt Loss_diff_. If the relationship between group and Wt Loss_diff_ is reduced after accounting for change in energy intake, then partial mediation will have been demonstrated.

## Discussion

Weight gain and obesity are the consequence of positive energy balance, which occurs when caloric intake exceeds calories expended [60]. Physical activity is the only aspect of energy expenditure that can be volitionally altered through activities such as exercise; yet, the importance of exercise in promoting and maintaining weight loss remains a complex issue. Although there is agreement that regular exercise plays an important role in general health, its exact role in the prevention of weight gain, weight loss and the prevention of regaining weight requires further study, despite its being widely prescribed for these purposes. The idea that current exercise recommendations for weight loss and weight management may stimulate compensatory mechanisms resulting in diminished weight loss is an important clinical and public health issue. However, there is a paucity of research into the mechanisms by which long-term exercise may promote increased energy intake or reduce spontaneous activity and energy expenditure. The significance of the E-MECHANIC trial is evidenced by this dearth of research. The trial will directly address calls for RCTs that comprehensively examine the dose–response relationship of exercise to weight loss and body composition [11].

The E-MECHANIC study will fill a major gap in the current literature. We will accomplish this by examining the role of exercise in weight loss using optimal measures of all aspects of energy balance (that is, energy intake, spontaneous activity, resting metabolic rate). To the best of our knowledge, no studies to date have been designed specifically to assess the effect of exercise dose on energy intake and expected versus actual weight loss. We expect that our findings in this trial will have significant public health implications because we will thoroughly evaluate whether and why current exercise recommendations for weight loss do not promote the level of weight loss expected based on the amount of exercise performed. We anticipate that the results of the E-MECHANIC study will provide important information related to the effects of exercise on energy intake and body weight by elucidating the mechanisms underlying exercise-induced weight compensation. Additionally, the participants in the E-MECHANIC study will be overweight and obese men and women, who are thus at risk for the negative health conditions associated with excess adiposity, namely, CVD and diabetes. Thus, the study sample precisely captures sedentary adults who may benefit most from regular exercise. These individuals are likely to be prescribed exercise regimens based on current guidelines. We anticipate that the results of the E-MECHANIC trial will clearly identify energy balance targets to prevent compensation and promote effective weight loss and management.

## Trial status

This trial is currently recruiting participants and was approximately 79% complete as of June 2014.

## Abbreviations

ACSM: American College of Sports Medicine; AEE: Activity energy expenditure; ATQ: Activity Temperament Questionnaire; BMA: Body Morph Assessment; BMI: Body mass index; CBC: Complete blood count; CBS: Current body size; CHBS: Compensatory Health Beliefs Scale; CVD: Cardiovascular disease; DLW: Doubly labeled water; DXA: Dual-energy X-ray absorptiometry; ECG: Electrocardiography; EE: Energy expenditure; EI: Eating Inventory; FNDDS: Food and Nutrient Database for Dietary Studies; HIPAA: Health Insurance Portability and Accountability Act; HR: Heart rate; IBS: Ideal body size; KKW: Kilocalories per kilogram of body weight per week; MAEDS: Multifactorial Assessment of Eating Disorders Symptoms; MET: Metabolic Equivalent of Task; mph: Miles per hour; OR: Orientation; PSQI: Pittsburgh Sleep Quality Inventory; RBS: Realistic body size; RCT: Randomized controlled trial; RI: Run-in; RMR: Resting metabolic weight; SV: Screening visit; TEE: Total energy expenditure; USDA: US Department of Agriculture; VAS: Visual Analogue Scale; YFAS: Yale Food Addiction Scale.

## Competing interests

The authors declare that they have no competing interests.

## Authors’ contributions

CAM was responsible for data collection and analysis and manuscript writing. WDJ was responsible for the conception and design of the study, data collection and analysis and manuscript writing. CPE was responsible for the conception and design of the study and critical revision of the manuscript. JCR was responsible for the conception and design of the study, data collection and analysis and critical revision of the manuscript. CTL and NMJ were responsible for the conception and design of the study and manuscript writing. SC and MH were responsible for data collection and analysis and critical revision of the manuscript. TSC and CKM were responsible for the conception and design of the study and manuscript writing. All authors will be responsible for conducting the trial. All authors read and approved the final version of the manuscript.
